# Characterization of Innate Immune Responses of Human Endothelial Cells Induced by *Porphyromonas gingivalis* and Their Derived Outer Membrane Vesicles

**DOI:** 10.3389/fcimb.2016.00139

**Published:** 2016-10-25

**Authors:** Meng-Hsuan Ho, Zhong-Mao Guo, Julio Chunga, J. Shawn Goodwin, Hua Xie

**Affiliations:** ^1^Oral Biology, School of Dentistry, Meharry Medical CollegeNashville, TN, USA; ^2^Department of Physiology, Meharry Medical CollegeNashville, TN, USA; ^3^Fisk UniversityNashville, TN, USA; ^4^Department of Biochemistry and Cancer Biology, Meharry Medical CollegeNashville, TN, USA

**Keywords:** *P. gingivalis*, outer membrane vesicles, atherosclerosis, IL-8, E-selectin

## Abstract

Atherosclerosis, a chronic inflammatory disease of the blood vessels, is one of the most common causes of morbidity and mortality world-wide. Involvement of *Porphyromonas gingivalis* in atherosclerosis is supported by observations from epidemiological, clinical, immunological, and molecular studies. Previously we reported that *P. gingivalis* vesicles have a much higher invasive efficiency than their originating cells. Here, we further compare the role of *P. gingivalis* cells and their vesicles in expression of chemoattractant proteins including CXCL1, CXCL2, and CXCL8, and adhesive molecules such as E-selectin in human umbilical vein endothelial cells (HUVECs). Both *P. gingivalis* 33277 cells and vesicles were able to up-regulate expression of these molecules, while the vesicles acted as more potent inducers of the inflammatory response associated with the development of atherosclerosis, consequently resulting in significant monocyte adhesion to a monolayer of HUVECs. Interestingly, we found that elevated expression of CXCL8 and E-selectin in endothelial cells induced by *P. gingivalis* correlated with the invasive ability of *P. gingivalis* cells and vesicles. Non-invasive bacterial cells and vesicles had no effect on expression of these genes. This study highlights the potential risk of *P. gingivalis* cells and vesicles in initiation of atherosclerosis and provides a potential target for the development of novel therapeutics against bacteria-associated atherosclerosis.

## Introduction

*Porphyromonas gingivalis*, a Gram-negative bacterium, is associated with chronic periodontitis and with several systemic diseases including atherosclerosis (Lamont and Jenkinson, 1998; Ximénez-Fyvie et al., 2000; Hajishengallis et al., 2012; Hussain et al., 2015). Recently, a keystone pathogen hypothesis regarding the pathogenesis of periodontitis has been proposed, suggesting that the presence of *P. gingivalis* in the oral cavity, even at low-abundance, is capable of disturbing host-microbial homeostasis and inducing periodontitis (Hajishengallis et al., 2011, 2012). Previous studies demonstrated that *P. gingivalis* disrupts tissue homeostasis through manipulation of innate immunity, including complement and proinflammatory cytokines (Hajishengallis and Lamont, 2014; Hajishengallis, 2015).

*P. gingivalis* vesicles originate from outer membrane blebbing and contain mostly outer membrane components including lipopolysaccharides and outer membrane proteins (Xie, 2015) and exhibit the primary features of this organism. In fact, we recently demonstrated that *P. gingivalis* vesicles exhibit much higher invasive efficiency than their originating bacterial cells, although it appears that both invasive processes involve a clathrin-mediated endocytic machinery (Ho et al., 2015, 2016). Previous studies suggested that the effect of *P. gingivalis* vesicles on the human immune response system is a complicated matter and not always consistent with those induced by *P. gingivalis* cells. Animal studies have demonstrated that *P. gingivalis* vesicles with strong immunogenicity were able to elicit *P. gingivalis*-specific IgG and IgA in the serum of intranasal vaccinated mice as well as IgA in saliva, whereas whole cells did not (Nakao et al., 2011; Bai et al., 2015). It was suggested that the increased antigenicity found in vesicles might result from the more concentrated immune-dominant determinants on the vesicles compared to *P. gingivalis* cell surfaces. In addition, *P. gingivalis* vesicles appeared to repress immune responses induced by IFN-γ. Expression of several genes involved in IFN-γ signal transduction, including genes encoding class II transactivator, Jak1, and Jak2, proteins required for expression of MHC class II molecules, were down-regulated in vascular endothelial cells in the presence of *P. gingivalis* vesicles (Srisatjaluk et al., 2002). Since MHC class II molecules are essential for antigen presentation, it is likely that inhibition of their expression facilitates *P. gingivalis*' escape from immune surveillance.

In the study presented here, built on our previous work on comparison of host cell invasion efficiencies of *P. gingivalis* cells and their vesicles, we further determined the ability of *P. gingivalis* to induce innate immune responses in human umbilical vein endothelial cells (HUVECs). We found that after exposure to *P. gingivalis* cells or vesicles, HUVECs selectively expressed inflammatory mediators including IL8 and endothelial-leukocyte adhesion molecules such as E-selectin, which resulted in monocyte adhesion to HUVECs. These findings represent insight into the molecule mechanisms of *P. gingivalis* associated-atherogenesis.

## Materials and methods

### Bacterial strains and vesicle preparation and quantification

*P. gingivalis* 33277 was grown from frozen stocks in TSB (trypticase soy broth) or on TSB blood agar plates supplemented with yeast extract (1 mg/mL), hemin (5 μg /mL), and menadione (1 μg/mL), and incubated at 37°C in an anaerobic chamber (85% N2, 10% H2, 5% CO2). *P. gingivalis* vesicles were prepared as previously described (Furuta et al., 2009). Briefly, *P. gingivalis* was grown to the late exponential phase and growth media were collected by centrifugation at 10,000 × g for 15 min at 4°C and filtered through 0.22-μm-pore-size filters (Cell Treat, MA, USA) to remove residual bacteria. Vesicles were collected by ultracentrifugation at 126,000 × g for 2 h at 4°C and resuspended in phosphate-buffered saline (PBS) containing 10% glycerol.

### Quantitation of *P. gingivalis* vesicles

Since quantifying vesicles by their protein or lipid content in weight represents the most common way to normalize data (Kulp and Kuehn, 2010), we quantitated OMVs using both protein and lipid assays. Proteins and lipids were extracted from vesicles in PBS using a BugBuster® Protein Extraction Reagent (Novagen, MA, USA). The quantity of OMV lipid was assessed using the fluorescent lipophilic dye FM4-64 as described (Macdonald and Kuehn, 2013), and was quantitated by titration of *P. gingivalis* lipopolysaccharide (LPS-PG, InvivoGen, San Diego, California). Protein concentrations were determined with a Bio-Rad Protein Assay Kit (Bio-Rad, CA, USA). Results revealed that 1 × 10^6^ *P. gingivalis* cells is equivalent to 100 ng protein or 3.6 μg lipid of vesicles. Thus, for *in vitro* infection experiments, 1 × 10^5^ host cells were exposed to 1 × 10^6^ *P. gingivalis* cells or vesicles with 100 ng protein.

### Treatment of endothelial cells with *P. gingivalis* 33277 and its vesicles

Umbilical vein endothelial cells (HUVECs) from American Type Culture Collection (ATCC, VA, USA), were cultured in specific media, according to the manufacturer's instructions. Prior to treatment, HUVECs (1 × 10^5^) were seeded and grown overnight in poly-L-lysine coated plates (CellTreat) at 37°C, 5% CO2, and then exposed to *P. gingivalis* 33277 (1 × 10^6^) or its vesicles (100 ng). The cytotoxicity of treatments was evaluated with a Pierce LDH Cytotoxicity Assay Kit (Thermo Scientific, MA, USA). There was no cytotoxicity detected under our experimental conditions.

### PCR array

After exposure to *P. gingivalis* 33277 cells or their vesicles, the HUVECs were immediately transferred to TRIzol (Thermo Scientific) to release RNA. The total RNA was purified using an RNeasy Mini Kit (QIAGEN, CA, USA). A total of 500 ng RNA was reverse-transcribed to cDNA with an RT2 First strand Kit (QIAGEN) and suspended with dd-H_2_O in a final solution of 111 μL. cDNAs were then subjected to a Human Antibacterial Response RT2 Profiler PCR Array (QIAGEN) that includes a set of optimized real-time PCR primer assays on 96-well-plates. Real-time PCR was run on a CFX96 Touch™ Real-Time PCR Detection System (Bio-Rad). Data were analyzed using a RT2 Profiler PCR Array Data Analysis (version 3.5, QIAGEN). RNA from three independent cultures was analyzed.

### Determination of *P. gingivalis* invasive ability using confocal microscopy

HUVECs, after exposed to *P. gingivalis* cells or their vesicles, were fixed with 2% formaldehyde in a Vascular Cell Basal Medium (ATCC) at room temperature for 10 min after treatments, permeabilized with 0.1% Triton X-100 for 10 min, and blocked with 10% horse serum in PBS for 1 h. The cells were then immunostained with polyclonal antibodies of *P. gingivalis* 33277, and anti-E-selectin monoclonal IgG (Santa Cruz Biotechnology, Texas, USA), followed by donkey anti-rabbit IgG conjugated Alex Fluor 546, chicken anti-mouse IgG conjugated Alex Fluor 488, or donkey anti-mouse IgG conjugated to Alex Fluor 546 (Thermo Scientific). Nuclei were stained with DAPI (Thermo Scientific). Confocal images were acquired using a Nikon A1R confocal microscope, and bacterial invasion abilities were determined by measuring intracellular florescence intensity in 10 random areas (5.6 × 5.6 μm) with an imaging software NIS-Elements AR 4.20.

### RNA isolation and qPCR

HUVECs treated with bacterial cells or vesicles were harvested by centrifugation and homogenized in Trizol Reagent (Thermo Scientific). The RNA in the supernatant was then purified using an RNeasy mini spin column (QIAGEN). RNA samples were digested on the column with RNase-free DNase. RT-PCR analysis was performed by using a QuantiTect SYBR Green RT-PCR Kit (QIAGEN) on the iCycler MyiQ™ Real-Time PCR detection system (Bio-Rad) according to the manufacturer's instructions. Primers are listed in Table S1. Amplification reactions consisted of a reverse transcription cycle at 50°C for 30 min, an initial activation at 95°C for 15 min, and 40 cycles of 94°C for 15 s, 58°C for 30 s, and 72°C for 30 s. The expression levels of the investigated genes for the test sample were determined relative to the untreated calibrator sample by using the comparative cycle threshold (Δ*C*_*T*_) method. The Δ*C*_*T*_ were calculated by subtracting the average *C*_*T*_-value of the test sample from the average *C*_*T*_-value of the calibrator sample, and were then used to calculate the ratio between the two by assuming 100% amplification efficiency. By loading the same amount of total RNA for any comparable samples, the Δ*C*_*T*_ represents the difference on gene expression between the samples.

### Enzyme-linked immunosorbent assay (ELISA)

ELISA was performed using a Human IL-8 Single Analyte ELISArray Kit (QIAGEN), according to the manufacturer's instruction. HUVECs were exposed to *P. gingivalis* 33277 cells or vesicles for 0, 2, 6, and 36 h. The culture media of HUVECs were collected and subjected to ELISA analysis. Concentration of IL-8 was determined using the standard curve of IL-8.

### Monocyte adhesion to HUVEC

HUVECs (5 × 10^4^/well) were seeded on 24-well plates and cultured for 2 days to reach a confluent monolayer. HUVECs were then co-cultured with *P. gingivalis* 33277 cells (5 × 10^6^) or vesicles (250 ng) for 20 h, and washed with phosphate-buffered saline (PBS) to remove unbound bacterial cells or vesicles. THP-1 cells were labeled with the fluorescent dye calcein AM (10 μM; Thermo Scientific) for 1 h, washed, and resuspended in RPMI medium containing 10% FBS. The labeled THP-1 cells (4 × 10^5^) were incubated with HUVEC monolayers for 2 h. After being washed with RPMI medium containing 10% FBS, adhesion of THP-1 cells to HUVEC was visualized under a Nikon TE2000-E immunofluorescence microscope. Fluorescence images were analyzed by imaging software NIS-Elements AR 4.20 to measure florescence intensity in 10 random areas (5.6 × 5.6 μm).

### Statistical analyses

A student's *t*-test was used to determine statistical significance of the differences in expression level of inflammatory genes in HUVECs in the presence or absence of *P. gingivalis* cells or vesicles. *P* < 0.05 was considered significant. Values are shown ±*SD* unless stated otherwise.

## Results

### Differential expression of inflammatory genes in HUVECs in response to *P. gingivalis* stimulation

Innate immune responses in HUVECs induced by *P. gingivalis* cells or vesicles were first examined using a PCR array that includes 84 key genes involved in innate immune response. The relative expression level of these genes in the HUVECs exposed to either *P. gingivalis* cells or vesicles was determined by comparing to those observed in HUVECs without any treatment. Three genes encoding CXCL1, CXCL2, and IL-8 were found to be the most highly expressed genes in HUVECs treated with vesicles derived from *P. gingivalis* 33277 for 2 h (Table 1). The gene encoding IL-8 was the only one, out of 84 inflammatory genes, that was slightly up-regulated in HUVECs exposed to 33277 cells.

**Table 1:** **Differential expression of inflammatory response related genes in HUVECs**.

**Genes**	**HUVECs treated with 3327 cells^a^**		**HUVECs treated with 3327 vesicles^b^**	
	**Fold change^c^**	***P*-value**	**Fold change^c^**	***P*-value**
CXCL1	1.075 ± 0.06	0.056	2.725 ± 1.03	0.022
CXCL2	1.045 ± 0.13	0.288	2.512 ± 0.66	0.008
IL-8	1.220 ± 0.12	0.018	3.406 ± 1.20	0.013

HUVECs (2.5 × 10^5^) were exposed to P. gingivalis 33277 cells (2.5 × 10^6^)

a or their vesicles. (250 ng)

b for 2 h. Gene expression was determined using Human Antibacterial Response. PCR Array, and fold change

c *of gene expression level is relative to that in HUVECs without any treatment*.

**Figure 1:**
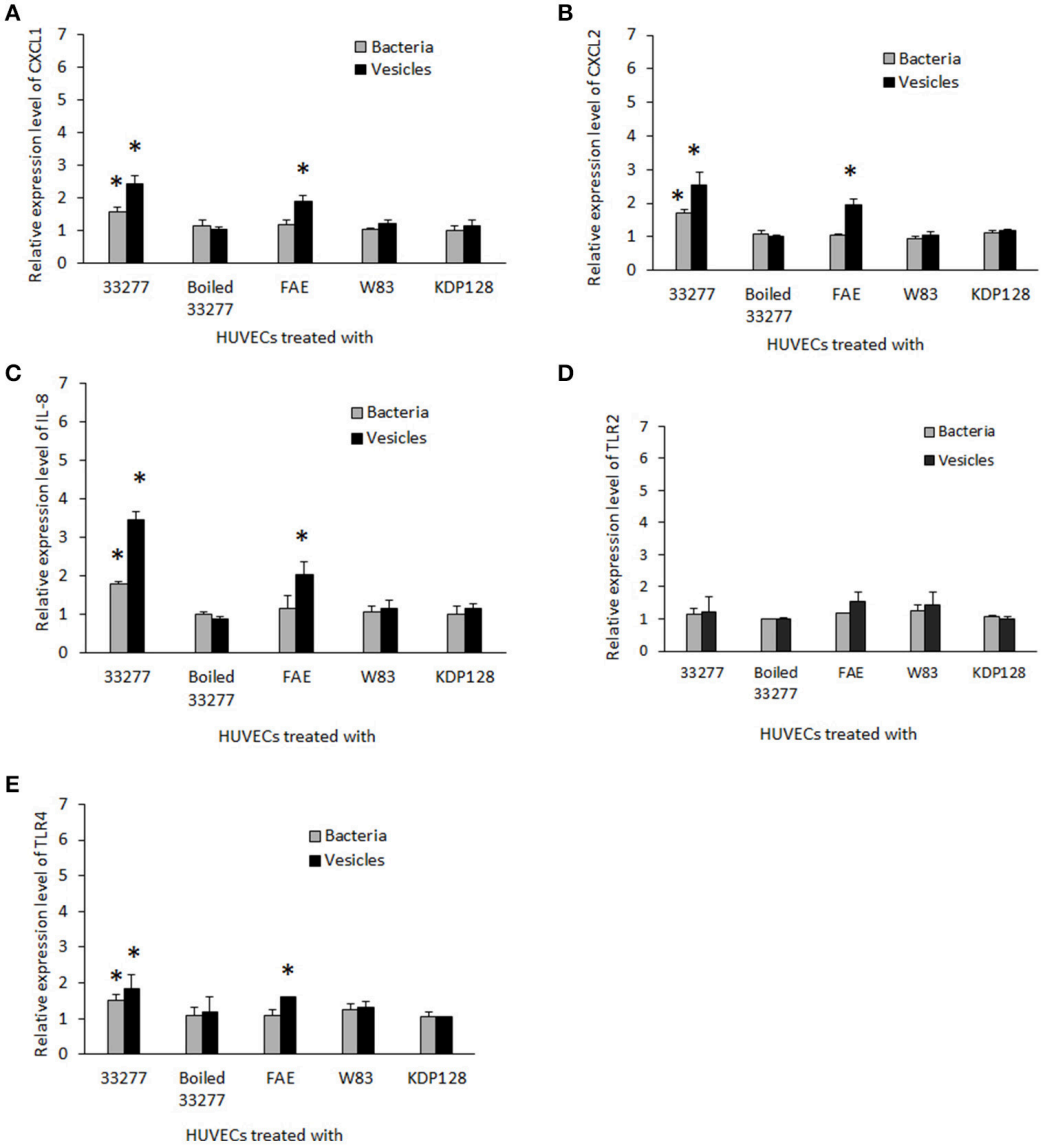
**Expression of inflammatory gene s in human endothelial cells in response to ***P. gingivalis*** infection**. HUVECs were infected with different *P. gingivalis* strains including wild type 33277, its isogenic *fimA* mutant (FAE), the isogenic ginginpain mutant (KDP128, *rgpA*^−^, *rgpB*^−^, and *kgp*^−^), heat treated 33277 and W83 strains, as well as the vesicles derived from these strains for 2h. mRNA levels of *cxcl-1* **(A)**, *cxcl2* **(B)**, *il-8* **(C)**, *tlr2* **(D)**, and *tlr4* **(E)** were determined in the infected HUVECs using qPCR. Expression level of each gene was normalized with that of Glyceraldehyde 3-phosphate dehydrogenase gene (*gapdh*). Each bar represents means of fold change with standard deviation of three biological replicates relative to that found in PBS treated HUVECs (1 unit). Asterisks indicate statistical significance of expression level in HUVECs treated and untreated with *P. gingivalis* (*P* < 0.05; *t*-test).

**Figure 2:**
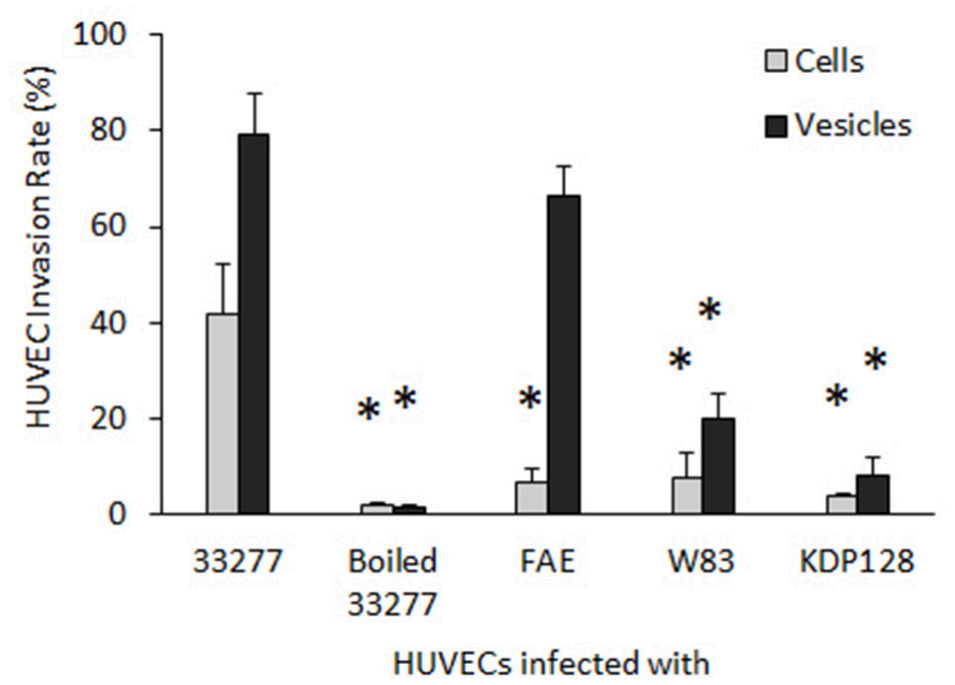
**Invasive activity of ***P. gingivalis*** cells and vesicles into HUVECs**. After exposed to *P. gingivalis* cells or vesicles, HUVECs were immune strained with *P. gingivalis* antibodies and analyzed under a confocal microscope. The number of HUVECs carrying intercellular *P. gingivalis* cells or vesicles (infection rate) was determined by counting the infected HUVECs in 30 random areas. Each bar represents the percentage of HUVECs with intercellular cells or vesicles. The SEs are indicated (*n* = 3). An asterisk indicates the statistical significance of invasive rates between *P. gingivalis* 33277 cells or vesicles and cells and vesicles of other *P. gingivalis* strains (*P* < 0.05; *t*-test).

**Figure 3:**
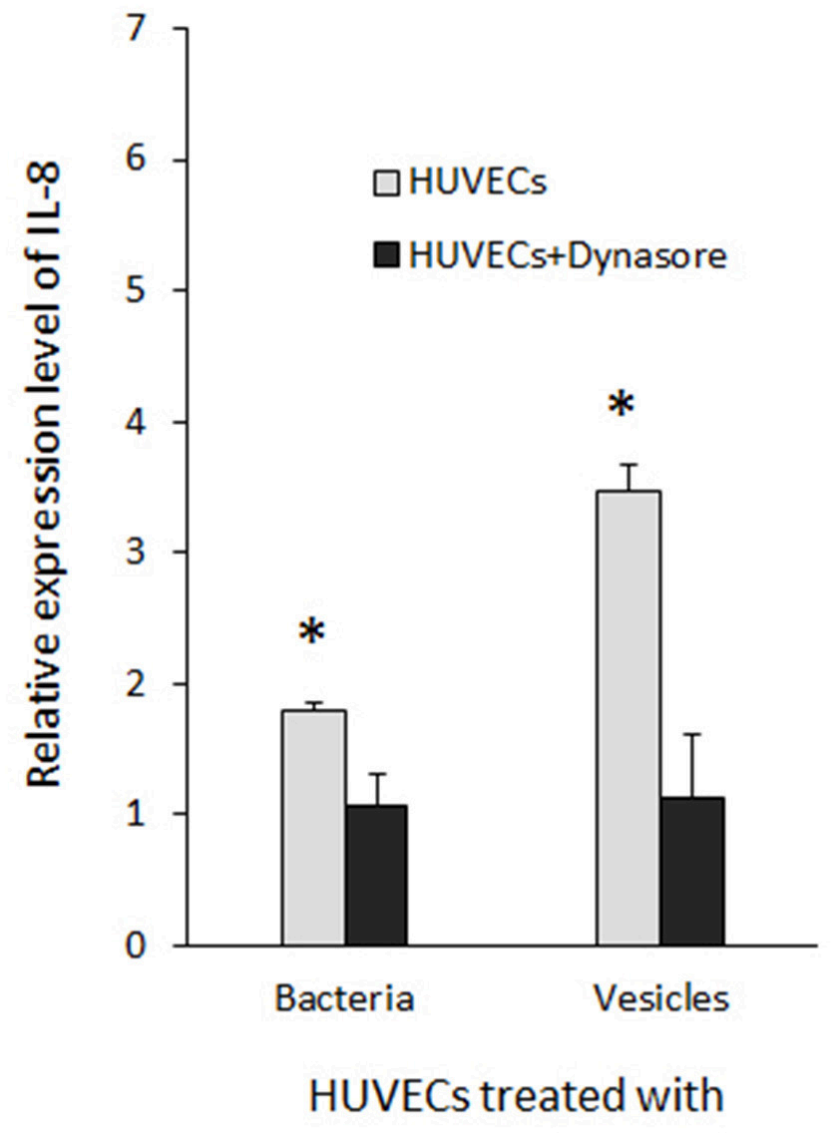
**Inhibition of ***P. gingivalis*** induced IL-8 expression by dynasore**. HUVECs were treated with dynasore (30 μM) prior to be exposed to *P. gingivalis* 33277 cells or vesicles. Expression level of IL-8 was determined using qRT-PCR and normalized with that of *gapdh*. Each bar represents means of fold change relatively to that found in HUVECs without *P. gingivalis* treatment (1 unit). Asterisks indicate the statistical significance of expression level in HUVECs treated and untreated with *P. gingivalis* (*P* < 0.05; *t*-test).

To assess the reliability of the PCR array results, we conducted qRT-PCR to measure mRNA levels for key genes involved in inflammatory responses individually using the identical total RNA samples used in the PCR array but different sets of primers (Table S1). Five inflammation related genes, including *cxcl1, cxcl2, cxcl8* (IL-8), *tlr-2*, and *tlr-4*, were tested. Ratios of the transcripts from untreated HUVECs to HUVECs treated with 33277 cells or vesicles, determined by the PCR array or by qRT-PCR of individual genes, were in a good concordance. Expression of *cxcl1, cxcl2*, and *IL8* were up-regulated ~2–3 fold in the vesicle-treated HUVECs compared to those in untreated cells (Figures 1A–C). Significant increase in the mRNA level of these genes was also observed in HUVECs treated with 33277 cells but at a lower degree. Up-regulated toll like receptor 4 was found in HUVECs treated with 33277 cells or vesicles, while expression of toll like receptor 2 was not altered in these cells (Figures 1D,E). Heat treatment of 33277 cells or vesicles abolished their activity to induce expression of *cxcl1, cxcl2*, and *IL8*, suggesting involvement of protein molecules. Other *P. gingivalis* strains including W83, the *fimA* mutant (FAE), and the gingipain triple mutant (KDP128, *rgpA*^−^, *rgpB*^−^, and *kgp*^−^) were also examined for their role in the induction. There was no significant alteration in expression of these inflammatory genes in the HUVECs treated with *P. gingivalis* cells or vesicles, except for the vesicles derived from the *fimA* mutant (FAE). It should be pointed out that although the *fimA* mutant loses its invasive activity, its vesicles are able to invade host cells without the FimA protein (Mantri et al., 2015). These data imply that the invasive ability of *P. gingivalis* is required for modulation of inflammatory gene expression. This is based on the observation that W83 cells and vesicles, the *fimA* mutant cells, the gingipain mutant cells and vesicles, and heat treated 33277 cells and vesicles showed little invasive activity (Figure 2) and were not able to significantly upregulate expression of the inflammatory genes tested. To confirm this assumption, HUVECs were pretreated with dynasore (30 μM), a potent inhibitor of *P. gingivalis* invasion (Ho et al., 2016). As expected, *P. gingivalis* 33277 cells and vesicles no long elicited expression of IL-8 in HUVECs (Figure 3).

**Figure 4:**
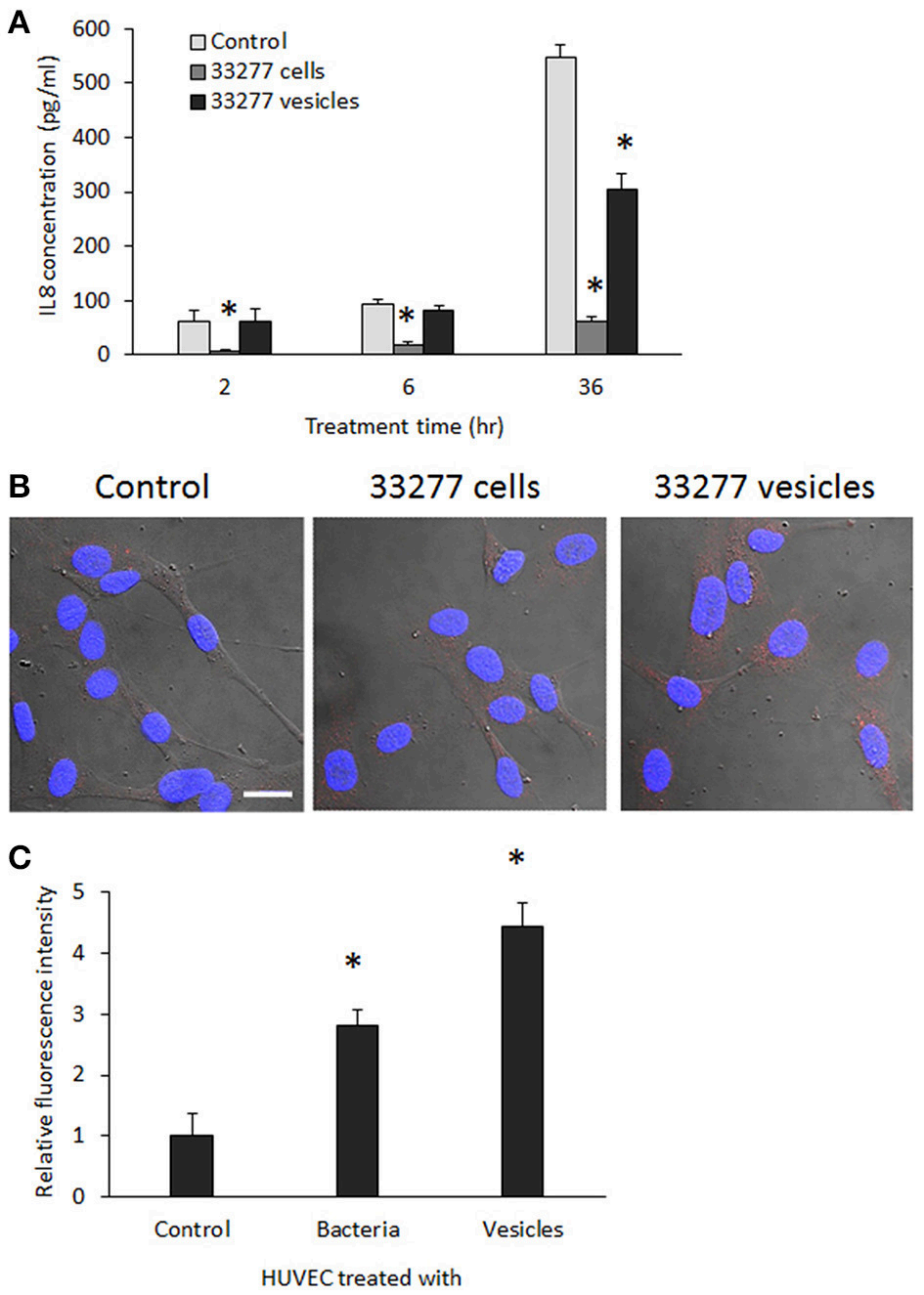
**Determination of IL-8 level in the culture supernatants and cytoplasm of HUVECs. (A)** HUVECs were exposed to *P. gingivalis* 33277 cells or vesicles for 2, 6, and 36 h, and culture supernatants collected. IL-1 level in the culture supernatants was measured using ELISA with a Human IL-8 Single Analyte ELISArray Kit (Qiagen). Each bar represents means of IL-8 concentration with standard deviation of three biological replicates. Asterisks indicate statistical significance of IL-8 concentration in the culture supernatants of HUVECs treated and untreated with *P. gingivalis* (*P* < 0.05; *t*-test). **(B)** Intracellular IL-8 was visualized in HUVECs using anti-E-selectin antibody and Alexa Fluor 546-conjugated secondary antibody (red) under a confocal microscope. Scale bar 20 μm. **(C)** Intracellular IL-8 was quantitated using an imaging software NIS-Elements AR 4.20. Asterisks indicate the statistical significance of florescence intensity in HUVECs treated and untreated with *P. gingivalis* (*P* < 0.05; *t*-test).

We then determined IL-8 level in the culture supernatant of HUVECs exposed to *P. gingivalis* 33277 cells or vesicles. IL-8 level was 90% less in the culture supernatant in the presence of 33277 cells compared to that found in the supernatant without 33277 cells, indicating degradation of IL-8 by the bacterial cells (Figure 4A). Previously, we showed that gingipains were enriched in *P. gingivalis* vesicles (Mantri et al., 2015), however, a much slower degradation of IL-8 was observed in the presence of 33277 vesicles, especially at 2 or 6 h exposure. We therefore speculate that membrane-associated gingipains have limited enzymatic activity.

IL-8 level in the cytoplasm of HUVECs was visualized using confocal microscopy. Significantly increased expression of IL-8 was found in HUVECs treated with *P. gingivalis* 33277 vesicles for 18 h (Figure 4B). Quantitation of IL-8 was conducted using imaging software NIS-Elements AR 4.20 to measure florescence intensity in 10 random areas (5.6 × 5.6 μm), and 2.5- and 4-fold increase of IL-8 was observed in HUVECs treated with 33277 cells or with the vesicles compared to that in untreated HUVECs (Figure 4C), suggesting that degradation does not occur in cytoplasm. It is likely that the HUVECs treated with 33277 vesicles constitutively release more IL-8, which may maintain a stable level of IL-8 in the micro-environment, although secreted IL-8 will eventually be degraded by extracellular *P. gingivalis* cells and vesicles.

### Induction of adhesion molecule expression in HUVECs by *P. gingivalis*

**Figure 5:**
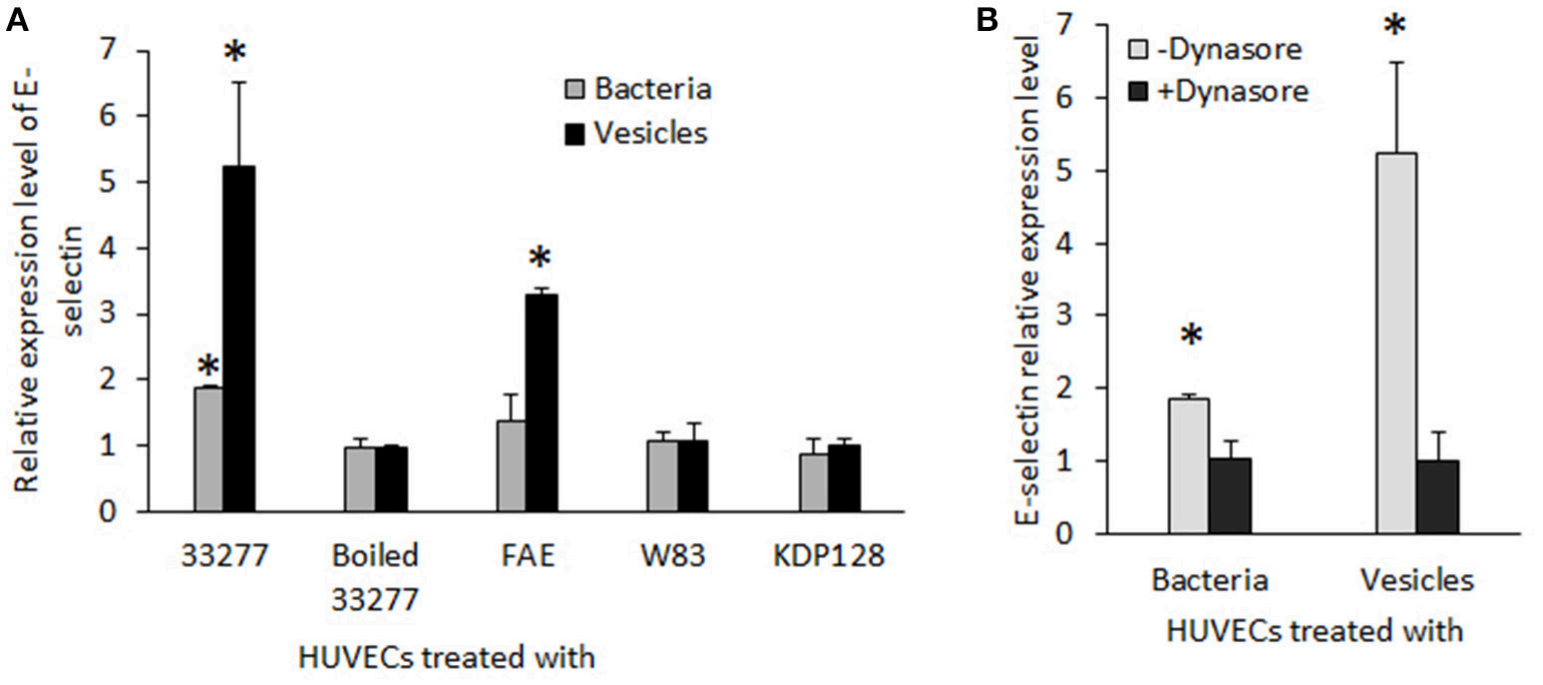
**Expression of E-selectin in HUVECs exposed to ***P. gingivalis*** strains. (A)** mRNA level of E-selectin in HUVECs in response to live *P. gingivalis* 33277, heat-treated 33277, the *fmA* mutant (FAE), the gingipain mutant (KDP128), heat treated 33277, and W83 strains and their derived vesicles was determined using qRT-PCR. **(B)** HUVECs were treated with dynasore (30 μM) prior to be exposed to *P. gingivalis* 33277 cells or vesicles. mRNA level of IL-8 was normalized with that of Glyceraldehyde 3-phosphate dehydrogenase gene (*gapdh*). Each bar represents means of fold change relative to that found in PBS treated HUVECs (1 unit). All experiments were repeated three times, and mean values are shown. The error bars indicate standard deviations. Asterisks indicate statistical significance of expression level in HUVECs treated and untreated with *P. gingivalis* (*P* < 0.05; *t*-test).

To determine the effect of *P. gingivalis* infection on expression of atherosclerosis-associated proteins, we measured expression of three adhesion molecules (E-selectin, VCAM1, and ICAM-1) in HUVECs at the transcriptional level using qRT-PCR. The results showed that mRNA of E-selectin was increased about 2 and 5 fold in the HUVECs treated with *P. gingivalis* 33277 cells or vesicles, respectively, compared to those detected in the HUVECs without any treatment (Figure 5A). This phenomenon of up-regulated adhesion molecules appears specific to E-selectin, as there was no alteration in mRNA levels of VCAM1 and ICAM-1 in HUVECs treated *P. gingivalis* (data not shown). A three-fold increase in mRNA level of E-selectin was also detected in the HUVECs treated with vesicles derived from FAE (the *fimA* mutant) but not the FAE cells. This effect was also not observed in the HUVECs treated with *P. gingivalis* W83, KDP128, or heat treated 33277, nor their vesicles. Not surprisingly, treatment of HUVECs with dynasore (30 μM) blocked *P. gingivalis* 33277-induced up-regulation of E-selectin (Figure 5B).

**Figure 6:**
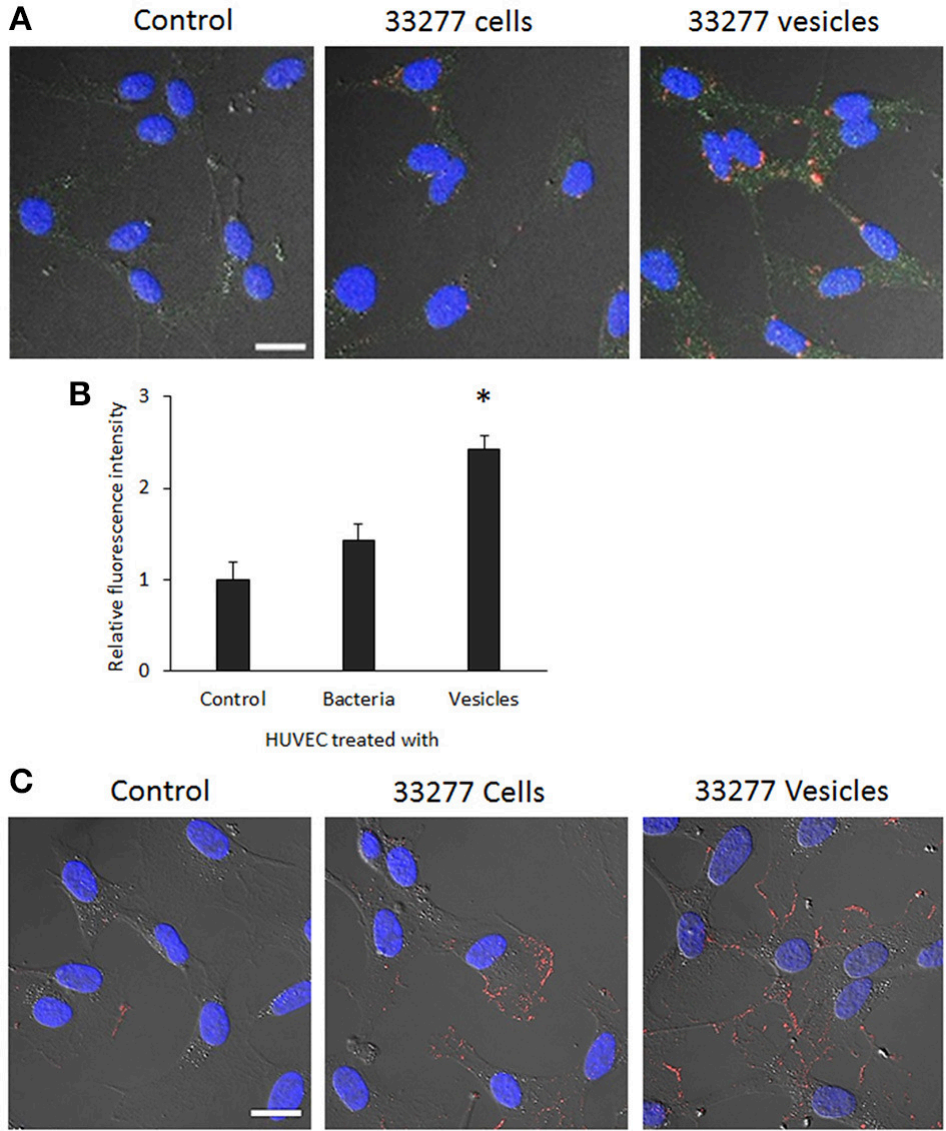
**Visualization of E-selectin in the cytoplasm and the surface of HUVECs with confocal microscopy. (A)** HUVECs were grown in a glass bottom dish for 16 h, and then infected with *P. gingivalis* 33277 cells or its vesicles. HUVECs were permeabilized. Internalized bacterial cells or vesicles were probed with anti-*P. gingivalis* polyclonal antibodies, visualized by Alexa Fluor 546-conjugated anti-rabbit IgG secondary antibody (red), E-selectin by anti-E-selectin antibody and Alexa Fluor 488-conjugated secondary antibody (green), and nucleus by DAPI (blue) Images are presented by differential interference contrast (DIC). **(B)** E-selectin level in the cytoplasm was determined using imaging software NIS-Elements AR 4.20. **(C)** Expression of E-selectin on the surface of HUVECs without permeabilization was visualized by anti-E-selectin antibody and Alexa Fluor 546-conjugated secondary antibody (red). Scale bar, 20 μm. An asterisk indicates the statistical significance of florescence intensity in HUVECs treated and untreated with *P. gingivalis* vesicles (*P* < 0.05 by *t*-test).

To determine if expression of E-selectin protein is elevated on the surface and/or in the cytoplasm of the HUVECs exposed to 33277 cells or vesicles, we performed confocal microscopic analysis using HUVECs with or without membrane permeabilization. Consistent with mRNA level of E-selectin, E-selectin protein was also significantly enhanced in cytoplasm of HUVECs when the cells were exposed to 33277 vesicles and permeabilized before immunofluorescence staining (Figures 6A,B). Additionally, we examined the surface expression of E-selectin on the HUVECs that were not permeabilized before staining. E-selectin expression was also enhanced on the surface of the HUVECs exposed to 33277 cells and to a much greater extent when exposed to 33277 vesicles (Figure 6C).

**Figure 7:**
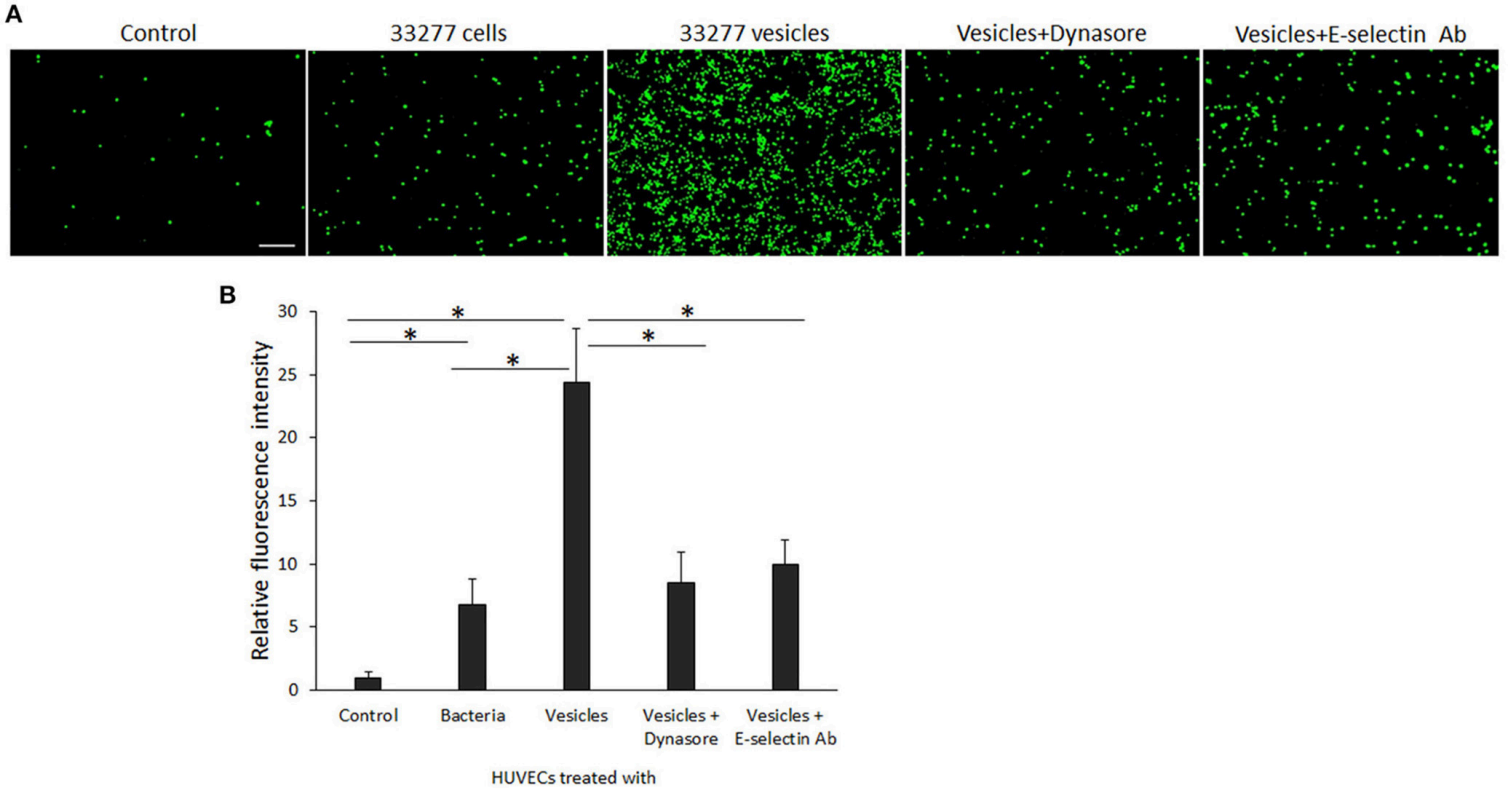
**Attachment of monocytes to ***P. gingivalis***-activated HUVECs. (A)** The calcein AM labeled THP-1 cells (green) were incubated with *P. gingivalis* 33277 cell- or vesicle-activated HUVEC monolayers for 2 h. After removing the unbound THP-1 cells, THP-1 adhesion was visualized under an immunofluorescence microscope. Scale bar, 100 μm. **(B)** Each bar represents the mean of fluorescence intensity in 10 random areas (5.6 × 5.6 μm) of immunofluorescence images. Comparison highlighted with an asterisk indicates a significant difference between two relative levels of florescence intensity detected on HUVEC monolayers cultured under different conditions (*P* < 0.05 by *t*-test).

E-selectin is known as endothelial-leukocyte adhesion molecule 1, and over-expression of E-selectin on activated endothelial cells leads to initial adhesion of leukocytes to endothelium. Using an adhesion assay, we found that attachment of monocytes (THP-1) on HUVEC monolayers was significantly enhanced. Compared to the attachment of THP-1 to HUVECs, 6- and 25-fold THP-1 cells were found on HUVEC monolayers treated with either *P. gingivalis* 33277 cells or vesicles, respectively (Figures 7A,B). We also examined THP-1binding to HUVEC monolayers treated with dynasore or anti E-selectin antibody. As shown in Figures 7, THP-1 attachment to the monolayers was significantly reduced when dynasore (30 μM) was added to the cultural media of HUVEC monolayers during exposure of *P. gingivalis* vesicles, or when the monolayers were treated with anti E-selectin antibody prior addition of THP-1. These results indicate that *P. gingivalis* invasion and its induced E-selectin expression are key events for promoting attachment of monocytes on HUVEC monolayers.

## Discussion

An association between *P. gingivalis* infection and atherosclerosis has been extensively investigated in *in vitro, ex vivo*, and animal models, which has made this bacterium a model of atherosclerosis initiated by microorganisms (Reyes et al., 2013). Previous studies have focused on intact *P. gingivalis* cells, based on the discovery of proteins and DNA of this bacterium in *ex vivo* samples. It was speculated that *P. gingivalis* may enter microvasculature following tooth brushing or other dental procedures, which may lead to transient bacteremia (Kinane et al., 2005; Iwai, 2009). However, it has not been confirmed if live cells of *P. gingivalis* cause low-grade inflammation in the walls of arterial vessels. Based on the recent findings, including the much more efficient invasive activity of *P. gingivalis* vesicles and the presence of vesicle-associated major outer membrane proteins, DNA, and RNA in the vesicles (Ho et al., 2015), we propose a novel concept that vesicles serve a significant role in atherogenesis, and they likely represent a “Trojan horse” to induce infections at secondary sites, such as in the walls of vessels, more so than intact *P. gingivalis* cells. In agreement with this concept, data presented here demonstrate that the vesicles are also more potent inducers of inflammation responses. Expression of IL-8 and E-selectin, at both mRNA and protein levels, was enhanced in HUVECs treated with *P. gingivlais* cells or vesicles, and more so for those exposed to vesicles. We also observed significantly enriched E-selectin on the surfaces of HUVECs treated with *P. gingivalis* 33277 vesicles, as well as highly induced monocyte adhesion to these HUVECs.

Interleukin (IL)-8 is a pro-inflammatory chemokine that belongs to the CXC subfamily and is also known as CXCL8 (Bacon et al., 2002). IL-8 plays an important role in inflammation, cancer, and cardiovascular disease through cell signaling and activation (Baggiolini and Clark-Lewis, 1992; Apostolakis et al., 2009; Chen et al., 2015). *P. gingivalis*-induced IL-8 production has been found in neutrophils, THP-1 cells, human periodontal ligament fibroblasts, and endothelial cells (Shelburne et al., 2007; Jayaprakash et al., 2014; Zhang and Li, 2015; Damgaard et al., 2016). However, reports on expression of IL-8 in human gingival epithelial cells in response to *P. gingivalis* infection have been conflicting (Takeuchi et al., 2013; Fujita et al., 2014; Yee et al., 2014; Savitri et al., 2015), and it was suggested that the different epithelial cell types used in those studies affect results. When comparing the abilities of *P. gingivalis* 33277 cells and the vesicles purified from the culture media to induce expression of IL-8 at the transcriptional level in HUVECs, we found that the mRNA level of IL-8 was higher in HUVECs treated with vesicles than that in HUVECs treated with 33277 cells, despite identical amounts of proteins and lipids were used. Interestingly, IL-8 accumulation in the culture media of HUVECs treated with 33277 vesicles was not significantly enhanced compared to that in the culture media of untreated HUVECs, while IL-8 level was dramatically decreased in the presence of *P. gingivalis* 33277 cells. Degradation of IL-8 has been reported previously, and Stathopoulou et al. showed that 100% degradation of IL-8 by live *P. gingivalis* cells could be reached after 30 min exposure (Stathopoulou et al., 2009). It was suggested that lysine gingipain (Kgp) is likely responsible for the degradation of IL-1 in culture supernatants (Stathopoulou et al., 2009; Jayaprakash et al., 2014). We reported earlier that gingipains were selectively enriched in *P. gingivalis* vesicles (Mantri et al., 2015). It is likely therefore that vesicle-associated gingipains are not as efficient as gingipains secreted by live *P. gingivalis* cells.

The role of Selectin in atherosclerosis is known for its ability to mediate rapid on-off interaction between monocytes and endothelium, which is called rolling adhesion (McEver and Zhu, 2010; Telen, 2014). Firm adhesion of monocytes to endothelium requires the presence of cytokines such as IL-8. Gerszten et al. revealed, using videomicroscopy, that IL-8 arrested the rolling monocytes on E-selectin-expressing endothelium. This likely involves recognition of β2 leukocyte integrin and chemokine receptor CXCR2 by IL-8 (Gerszten et al., 1999). It should be pointed out that CXCR2 is a receptor for both CXCL1 and IL-8; presumably up-regulation of CXCL1 may compensate degradation of IL-8 by gingipains or have a synergistic effect in monocyte adhesion.

One striking finding of this work is that mechanisms contributing to up-regulation of IL-8 and E-selectin in HUVECs appear to involve the invasive ability of *P. gingivalis* cells and vesicles. Previous studies have shown an involvement of a TonB-dependent receptor (RagB), LPS, and Fimbria-dependent activation of IL-8 in primary human monocytes (Hutcherson et al., 2015), human oral keratinocytes (Luo et al., 2012), and human aortic endothelial cells (Takahashi et al., 2006). Expression of E-selectin in human endothelial cells is also up-regulated by *P. gingivalis* penta-acylated lipid A and fimbrial proteins (Chou et al., 2005; Reife et al., 2006; Takahashi et al., 2006). We demonstrate here that the invasive ability of *P. gingivalis* also contributes to elevated IL-8 and E-selectin expression in human endothelial cells, which is in agreement with an observation by Takahashi et al. that only invasive *P. gingivalis* strains induced production of IL-8 and E-selectin in human aortic endothelial cells (Takahashi et al., 2006). Although the mechanisms have not been clarified, our data indicate that a dynamin-mediated endocytosis is required for eliciting expression of IL-8 and E-selectin. This conclusion is based on our finding that dynasore, an inhibitor of dynamin-mediated endocytosis (Macia et al., 2006), completely blocked the ability of *P. gingivalis* cells and vesicles to induce IL-8, and E-selectin expression. Collectively, our findings provide evidence that the invasive *P. gingivais* strains, especially their vesicles, are capable of inducing the production of pro-inflammatory and adhesive molecules that are hallmarks of atherosclerosis. Therefore, identification of agents to inhibit *P. gingivalis* invasion is likely an efficient therapeutic approach for intervention of the bacteria-associated atherosclerosis.

## Author contributions

Conceived and designed the experiments: HX. Performed the experiments: MH and JC. Analyzed the data: ZG and HX. Contributed reagents/materials/analysis tools: JSG and HX. Wrote the paper: MH, ZG, JSG, and HX.

### Conflict of interest statement

The authors declare that the research was conducted in the absence of any commercial or financial relationships that could be construed as a potential conflict of interest.
