# Supplementary material for: Characterization of Innate Immune Responses of Human Endothelial Cells Induced by Porphyromonas gingivalis and Their Derived Outer Membrane Vesicles
Source: Front Cell Infect Microbiol. 2016 Oct 25;6:139. doi: 10.3389/fcimb.2016.00139 (PMC5078693; doi:10.3389/fcimb.2016.00139)
Supplement: Supplementary file 1 [file Table1.PDF]

Table S1. Oligonucleotide primers used in this study

Gene	Primer name	Primer sequences (5'-3')
Glyceraldehyde 3-phosphate	GAPDH F	GGTGGTCTCCTCTGACTTCAACA
dehydrogenase (GAPDH)	GAPDH R	GTTGCTGTAGCCAAATTCGTTGT
Toll-Like Receptor 2 (TLR2)	TLR2 F	GGGTCATCATCAGCCTCTCC
	TLR2 R	AGGTCACTGTTGCTAATGTAGGTG
Toll-Like Receptor 4 (TLR4)	TLR4 F	CAGAGTTGCTTTCAATGGCATC
	TLR4 R	AGACTGTAATCAAGAACCTGGAGG
Chemokine (C-X-C Motif)	CXCL1 F	GCAGCAGGAGCGTCCGTGGC
Ligand 1 (CXCL1)	CXCL1 R	CAGTTGGATTTGTCACTGTTCAGCAT
Chemokine (C-X-C Motif)	CXCL2 F	TCACCTCAAGAACATCCAAAGTGTG
Ligand 2(CXCL2)	CXCL2 R	CTTCAGGAACAGCCACCAATAAGC
Interleukin 8 (IL-8)	IL-8 F	TTCTAGGACAAGAGCCAGGAAG
	IL-8 R	GGGTGGAAAGGTTTGGAGTATG
Intercellular Adhesion	ICAM-1 F	GACTCCAATGTGCCAGGCTT
Molecule 1 (ICAM-1)	ICAM-1 R	TAGGTGCCCTCAAGATCTCG
Vascular cell adhesion	VCAM-1 F	GATACAACCGTCTTGGTCAGCCC
molecule 1 (VCAM-1)	VCAM-1 R	CAGTTGAAGGATGCGGGAGTATATG
E-selectin	E-selectin F	AGAGGTTCCTTCCTGCCAAG
	E-selectin R	CAGAGCCATTGAGCGTCCAT
